# mettannotator: a comprehensive and scalable Nextflow annotation pipeline for prokaryotic assemblies

**DOI:** 10.1093/bioinformatics/btaf037

**Published:** 2025-01-24

**Authors:** Tatiana A Gurbich, Martin Beracochea, Nishadi H De Silva, Robert D Finn

**Affiliations:** European Molecular Biology Laboratory, European Bioinformatics Institute, Wellcome Genome Campus, Cambridge CB10 1SD, United Kingdom; European Molecular Biology Laboratory, European Bioinformatics Institute, Wellcome Genome Campus, Cambridge CB10 1SD, United Kingdom; European Molecular Biology Laboratory, European Bioinformatics Institute, Wellcome Genome Campus, Cambridge CB10 1SD, United Kingdom; European Molecular Biology Laboratory, European Bioinformatics Institute, Wellcome Genome Campus, Cambridge CB10 1SD, United Kingdom

## Abstract

**Summary:**

In recent years, there has been a surge in prokaryotic genome assemblies, coming from both isolated organisms and environmental samples. These assemblies often include novel species that are poorly represented in reference databases creating a need for a tool that can annotate both well-described and novel taxa, and can run at scale. Here, we present *mettannotator—*a comprehensive, scalable Nextflow pipeline for prokaryotic genome annotation that identifies coding and noncoding regions, predicts protein functions, including antimicrobial resistance, and delineates gene clusters. The pipeline summarizes these results in a GFF (General Feature Format) file that can be easily utilized in downstream analysis or visualized using common genome browsers. Here, we show how it works on 200 genomes from 29 prokaryotic phyla, including isolate genomes and known and novel metagenome-assembled genomes, and present metrics on its performance in comparison to other tools.

**Availability and implementation:**

The pipeline is written in Nextflow and Python and published under an open source Apache 2.0 licence. Instructions and source code can be accessed at https://github.com/EBI-Metagenomics/mettannotator. The pipeline is also available on WorkflowHub: https://workflowhub.eu/workflows/1069.

## 1 Introduction

The rapid rise of affordable high-throughput sequencing has resulted in a deluge of microbial genomes from isolates, single-cell sequencing and metagenomes (Hunt *et al.* 2024). While there are numerous genome annotation tools available, it is not always easy to install them or run them at scale–a major drawback for teams without bioinformatics expertise. Furthermore, while many pipelines hone in on the annotation of individual proteins, there is lesser focus on the identification of larger gene clusters which provide contextual information, or the systematic annotation of antimicrobial resistance genes (Haft *et al.* 2024). Annotation of novel genomes presents an additional challenge as these genomes are less likely to be represented in reference databases and, therefore, are likely to have less functional information computationally transferred onto them.

To address these issues, we have developed a comprehensive pipeline—*mettannotator*—that combines existing tools and custom scripts to perform both structural (demarcating genomic elements) and functional (assigning functions to genomic elements) annotation of prokaryotic genomes. By using a diverse set of annotation tools, the pipeline can handle genomes even without a species-level taxonomic label. In addition to using several reference databases, *mettannotator* builds upon established annotation frameworks used in UniProt (Bateman *et al.* 2023) to assign function to unannotated proteins. It also predicts larger genomic regions such as biosynthetic gene clusters, anti-phage defence systems, and putative polysaccharide utilization loci, and consolidates all annotations into a single GFF file. Implemented in Nextflow and following the best practices established by the nf-core community (Di Tommaso *et al.* 2017, Ewels *et al.* 2020), *mettannotator* adheres to the FAIR principles (Findable, Accessible, Interoperable, and Reusable) (Wilkinson *et al.* 2016). It is fully containerized, making it portable and easily deployable, scales well, is versioned (ensuring annotation provenance), and is discoverable. The pipeline can be run on isolate and metagenome-assembled genomes (MAGs), both bacterial and archaeal, and is now the unified annotation pipeline used by Ensembl Bacteria and MGnify (Gurbich *et al.* 2023, Harrison *et al.* 2024). We present the results of our pipeline on 200 genomes and compare it to other prokaryotic annotation packages.

## 2 Pipeline description

### 2.1 Installation and dependencies

Nextflow and Singularity/Apptainer or Docker are the only prerequisites for running *mettannotator*. Databases (total size 182 GB) are downloaded automatically during the first execution of the pipeline unless provided by the user. Users can also provide different database versions than the default ones supported by the pipeline. A minimum of 12 GB of RAM and 8 CPUs are required to run *mettannotator*.

### 2.2 Input files

The pipeline takes a single comma-separated text file as input which can include one or many genomes to be analyzed. For each genome, it is required to provide:

- a prefix, which will be used to name the result files and in the locus tags in the GFF file
- a path to the genome assembly in FASTA format (ideally oriented to start at *dnaA* or *repA*)
- a valid NCBI TaxId for the lowest known taxonomic level.

### 2.3 Annotation workflow

**Figure 1.**
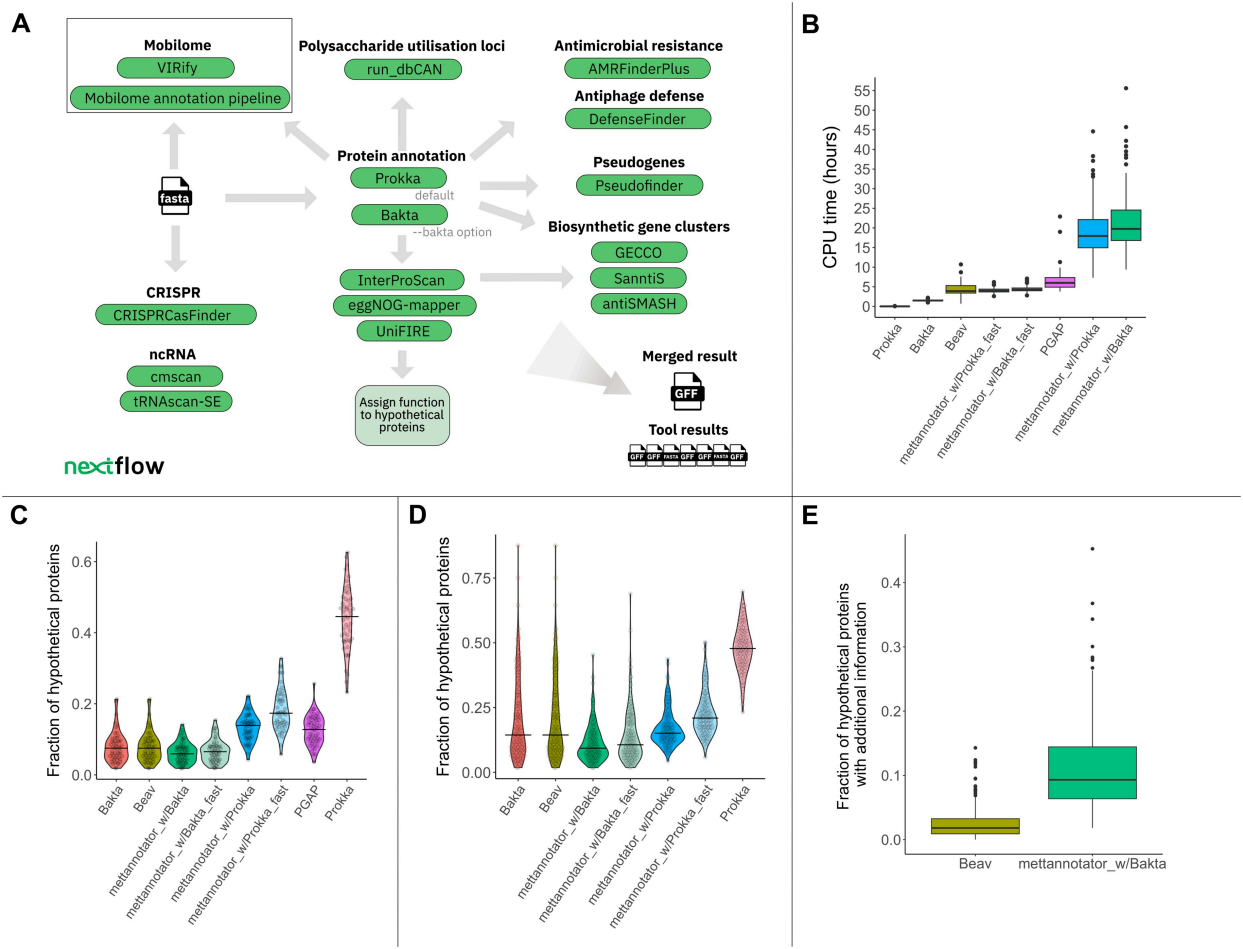
(A) The *mettannotator* workflow schema. The pipeline outputs the results of the individual tools as well as a merged GFF file. The mobilome annotation is not currently integrated into the Nextflow pipeline but can be executed separately (https://github.com/EBI-Metagenomics/mobilome-annotation-pipeline; Rangel-Pineros *et al.* 2023). The results can be added to the *mettannotator* GFF output file using the post-processing script provided. (B) CPU time per genome. *Mettannotator* (with Bakta and with Prokka as the gene caller, with and without the ‘—fast’ flag) and Prokka were run on 200 genomes, Bakta and Beav on 194 bacterial genomes, and PGAP on 51 genomes with known species-level taxonomy confirmed by PGAP. *Mettannotator* was faster than both PGAP and Beav when executed in the fast mode. When executed without the ‘—fast’ flag, *mettannotator* took longer than other tools to complete. (C) Fraction of hypothetical proteins out of all detected CDS, calculated for the 51 genomes that were annotated by PGAP demonstrating tool performance on genomes with known and confirmed species-level taxonomy. Horizontal bars indicate the median value. *Mettannotator* with Bakta as the gene caller has the lowest fraction of hypothetical proteins compared to other tools. (D) Fraction of hypothetical proteins out of all detected CDS. 194 bacterial genomes were used as inputs. When genomes from novel taxa are added to the analysis, the difference between *mettannotator* and other tools is more pronounced. (E) Fraction of proteins that are labeled as ‘hypothetical’ but have additional information available in the GFF file as reported by Beav and *mettannotator* (run in normal mode with Bakta as the gene caller). Both tools were run on 194 bacterial genomes. The tools provide additional functional information based on the results of the pipeline components that are either not used as a source for the ‘product’ field or were insufficient to assert a function. However, this additional information can help the user identify the putative function of a protein in cases where the product field is functionally unassigned.

The workflow schema is shown in Fig. 1A. The *mettannotator* pipeline offers the user a choice between two well established tools to perform gene prediction and initial annotation–Prokka (Seemann 2014), the default choice, and Bakta (Schwengers *et al.* 2021). Since Bakta is only intended for bacterial genome annotation, *mettannotator* determines the domain automatically based on the provided TaxId and always uses Prokka to annotate archaeal genomes. Potential pseudogenes produced by either Prokka or Bakta are identified and labeled by Pseudofinder (Syberg-Olsen *et al.* 2022).

Functional information provided by Prokka or Bakta is supplemented by InterProScan (Jones *et al.* 2014), eggNOG-mapper (Cantalapiedra *et al.* 2021), and UniFIRE, the UniProt Functional annotation Inference Rule Engine (https://gitlab.ebi.ac.uk/uniprot-public/unifire). Running UniFIRE allows *mettannotator* to deploy the automated function prediction system used by UniProt based on three sets of UniProt rules: curated UniRule and UniRule-PIRSR, and automatic, rule-mining-based ARBA (Saidi *et al.* 2017, Chen *et al.* 2019, MacDougall *et al.* 2020). The output of InterProScan includes matches to the AntiFam database (Eberhardt *et al.* 2012), which is a collection of families of spurious open reading frames. *Mettannotator* discards proteins that have an AntiFam match from the final annotation file.

Any proteins that are labeled as *hypothetical* by Prokka or Bakta are further processed to select a probable function from the outputs of InterProScan, UniFIRE, and eggNOG-mapper. The function assignment logic and prioritization of data sources are shown in Supplementary Fig. S1. When the functional assignment is possible, the ‘product’ field in the final GFF file produced by *mettannotator* is overwritten to reflect the detected function.

To generate a comprehensive annotation beyond individual protein function assignment, *mettannotator* delineates biosynthetic gene clusters (Medema *et al.* 2011, Carroll *et al.* 2021, Sanchez *et al.* 2023), identifies putative polysaccharide utilization loci (Zheng *et al.* 2023), anti-phage defence systems (Tesson *et al.* 2022), antimicrobial resistance genes (Feldgarden *et al.* 2021), CRISPR arrays (Couvin *et al.* 2018), and noncoding RNA (Nawrocki and Eddy 2013, Chan and Lowe 2019, Kalvari *et al.* 2021) (Fig. 1A). Versions of all tools and databases are listed in the README file in the GitHub repository and are reported by the pipeline in the MultiQC (Ewels *et al.* 2016) summary report.

Nextflow allows for seamless parallel annotation of multiple genomes, monitoring and re-starting the pipeline from any point of failure. At the end, the pipeline parses the results of each step and consolidates them into a final GFF file per genome.

*Mettannotator* also provides a convenient option to generate quick, draft annotations by using the ‘—fast’ flag. These are less in-depth, but take a fraction of the time by skipping InterProScan, UniFIRE, and SanntiS predictions. Functions of fewer proteins are resolved when the ‘—fast’ flag is used as eggNOG-mapper becomes the only additional source of information for the ‘product’ field.

### 2.4 Output files

M*ettannotator* produces a GFF file with results from all tools merged. The ninth column of the file contains carefully chosen key-value pairs to report the salient conclusions from each tool. Notably, we include KEGG orthology identifiers (Kanehisa *et al.* 2017), Gene Ontology (GO) terms (Thomas 2017), and Chemical Entities of Biological Interest (ChEBI) identifiers (Degtyarenko *et al.* 2008, Morgat *et al.* 2020, Bansal *et al.* 2022) parsed from eggNOG-mapper and UniFIRE outputs.

Annotations are visualized using the PyCirclize package (https://github.com/moshi4/pyCirclize) to produce Circos plots (Krzywinski *et al.* 2009) for genomes with up to 50 contigs. The pipeline output includes key files from each tool, organized in a structured directory system for each genome.

## 3 Performance evaluation

### 3.1 Test dataset and tools

We evaluated *mettannotator* against existing tools on a dataset that included 200 genomes: *E.coli* O26: H11 str. 11368 (GCA_000091005.1) (Ogura *et al.* 2009) and 199 genomes from MGnify (Gurbich *et al.* 2023), both MAGs and isolates, bacteria and archaea, from six different biomes, including host-associated and environmental ones. These genomes covered a wide range of taxa, including uncharacterized ones, and had different levels of completeness, contamination, and contiguity (Supplementary Table S1, Supplementary Methods).

We annotated this dataset using *mettannotator* v.1.4.0 (with Prokka and with Bakta as the base annotators, with and without the—fast flag), Bakta v1.9.3, Prokka v.1.14.6, PGAP v.2024-04-27.build7426 (Li *et al.* 2021, Haft *et al.* 2024), and Beav v1.3.0 (Jung *et al.* 2024). Bakta, Prokka, and PGAP were chosen for their wide usage and Beav was included due to its similar workflow (workflow comparison is shown in Supplementary Table S2). The tool execution parameters and the introduced filters and constraints are described in Supplementary Methods.

### 3.2 Performance results

First, we compared per-genome CPU time between tools (Fig. 1B). With the ‘—fast’ flag (skipping InterProScan, UniFIRE and SanntiS), *mettannotator* averaged 4.39 h with Bakta as the gene caller and 4.07 h with Prokka, faster than all tools except standalone Bakta and Prokka. The full version of *mettannotator* consumed more resources, averaging 21.20 h with Bakta and 19.07 h with Prokka as the gene callers. The most resource-intensive steps were InterProScan and UniFIRE (Supplementary Fig. S2), which are used by *mettannotator* to identify functions of unannotated proteins.

To measure the wall-clock time and provide guidance to end users, we annotated eight MGnify MAGs with *mettannotator* in both normal and fast modes and compared with Bakta v1.9.3 using its default settings. The benchmark was performed on a virtual machine equipped with 32 vCPUs (Intel Xeon Cascade Lake 2.5 GHz) and 128 GB of RAM.

Bakta averaged 16 min ± 6 min per genome. In comparison, *mettannotator* with Prokka in normal mode averaged 64 min ± 14 min, reduced to 12 min ± 5 min in fast mode. M*ettannotator* with Bakta in normal mode resulted in the longest average wall-clock time of 97 min ± 14 min, reduced to 41 min ± 8 min in fast mode.

To compare annotation results between the tools, we evaluated the fraction of unannotated, or ‘hypothetical’, proteins per genome. We classified any CDS labeled as ‘hypothetical protein’ or ‘uncharacterized protein’ in the final GFF file as hypothetical. While some of these proteins may have additional annotations, the tool could not assign a product due to either a lack of a high-quality database match or the specifics of the annotation algorithm.

All tools, except for Prokka, produced few hypothetical proteins (median < 20%) when executed on a set of 51 genomes with known species-level taxonomy and high level of completeness [mean = 95.65% as calculated by CheckM (Parks *et al.* 2015)] (Fig. 1C). *Mettannotator* had the lowest fraction of hypothetical proteins out of all tools when using Bakta as the gene caller (median = 5.9%).

The difference between tools was more pronounced on 194 bacterial genomes that included unknown taxa (PGAP was omitted due to its species-level taxonomy requirement) (Fig. 1D). *Mettannotator*, when run with Bakta, had the fewest hypothetical proteins (median = 9.3%). The difference was even greater on genomes with no assigned species name or poorly known taxonomy (Supplementary Fig. S3), highlighting *mettannotator’*s effectiveness in annotating novel genomes. The majority of hypothetical proteins relabeled by *mettannotator* inherited function from InterPro (Supplementary Fig. S4).

We examined additional functional information provided by Beav and *mettannotator* but not included in the product field (Fig. 1E, Supplementary Methods). *Mettannotator* had a higher fraction of proteins with extra annotations, indicating that although in some cases we are not able to assign a function, additional information provided, such as, e.g. an antimicrobial or antiviral defence annotation, can offer insights into potential protein roles.

Lastly, we evaluated the annotation quality by annotating a well-characterized biosynthetic locus using *mettannotator* (with Bakta and Prokka as the initial annotation tools, in fast and regular mode), Bakta, Prokka, PGAP, and Beav (Supplementary Methods, Supplementary Table S3). We evaluated the accuracy of gene boundary prediction, pseudogene detection, and gene name and product assignment. The quality of annotations produced by *mettannotator* in all four modes was comparable to that of other tools with all tools reliably assigning gene products to all genes in the cluster (Supplementary Results, Supplementary Table S3, and Supplementary Fig. S5).

## Supplementary Material

btaf037_Supplementary_Data

## Data Availability

The pipeline is freely available at https://github.com/EBI-Metagenomics/mettannotator.
