# Supplementary material for: mettannotator: a comprehensive and scalable Nextflow annotation pipeline for prokaryotic assemblies
Source: Bioinformatics. 2025 Jan 24;41(2):btaf037. doi: 10.1093/bioinformatics/btaf037 (PMC11842068; doi:10.1093/bioinformatics/btaf037)
Supplement: btaf037_Supplementary_Data [file btaf037_supplementary_data.zip › Supplementary methods, results and figures.docx]

**Supplementary methods**

**Test dataset**

Completeness and contamination of the 200 genomes included in the test dataset were calculated using CheckM v1.1.3 (Parks et al. 2015). Taxonomy was determined using GTDB-Tk v.2.4.0 with reference database release 220 (Chaumeil et al. 2020). TaxId for the *mettannotator* input file was determined using CAT v.5.3 (von Meijenfeldt et al. 2019). *E.coli* (GCA_000091005.1) taxonomy was manually replaced as CAT only classified this genome down to the superkingdom level.

The dataset includes 159 MAGs and 41 isolate genomes. The average genome completeness is 89.2% and maximum contamination is 5.13%. The number of contigs ranges from 1 to 889 and the genome length ranges from 583.5 Kb to 10.6 Mb. Twenty-nine prokaryotic phyla are represented in the dataset.

The genomes originated from six biomes (MGnify genome catalogue versions are in parentheses): human gut (v2.0.2), human oral (v1.0.1), cow rumen (v1.0.1), fish gut (v2.0), marine (v2.0), and chicken gut (v1.0.1).

**Tool execution**

*mettannotator*

*Mettannotator* was run in 4 modes - with Prokka (Seemann 2014) and with Bakta (Schwengers et al. 2021) as the gene callers and base annotators and with and without the --fast flag. To compute CPU time, we executed *mettannotator* one genome at a time and used the CPU time reported by Nextflow.

PGAP

PGAP (Haft et al. 2024; Li et al. 2021) requires that the user provides either the genus or the species name as the organism name when running the annotation workflow. To determine taxonomies that would pass PGAP’s checks, we first executed PGAP on each test genome with the --taxcheck-only flag enabled. Out of 200 genomes, PGAP found a “best match” for 137 genomes. We reran PGAP on these genomes with the --taxcheck-only flag enabled providing PGAP’s highest species-level taxonomy match from the results of the previous run as the organism name. Taxonomy of 42 genomes was confirmed by PGAP with high confidence, further 9 genomes were confirmed with low confidence, and two MAGs, MGYG000299175 and MGYG000299211, were marked as contaminated by PGAP but reported as having high confidence. We included all of the genomes with confirmed taxonomy, 51 in total, in the annotation input dataset. We were not able to improve PGAP’s taxonomy confirmation result when we only provided the genus name rather than the species name for any of the genomes in our dataset. Consequently, all genomes that were retained for further analysis were given species-level organism names.

The dataset annotated by PGAP comprises 25 MAGs and 26 isolate genomes. It includes 22 genomes from the human gut, 8 from the human oral cavity, 7 from marine environments, 7 from the fish gut, 6 from the chicken gut, and 1 from the cow rumen. The average genome completeness is 95.65% (Supplementary Table 1).

Genomes were annotated using 16 CPUs and 50 GB of RAM and with --no-internet, --no-self-update and --ignore-all-errors flags enabled. The Docker container was downloaded prior to execution and no tax check was performed during the annotation process.

Bakta

Bakta was run on all bacterial genomes in the test dataset (194 in total) using 8 CPUs and 25 GB of RAM per genome. Two genomes (MGYG000298952 and MGYG000298476) were annotated with the --skip-crispr flag enabled due to this portion of the workflow failing.

Prokka

Prokka was run on all archaeal and bacterial genomes (200 in total) using 8 CPUs and 25 GB of RAM per genome.

Beav

Beav (Jung et al. 2024) was run on all bacterial genomes with 8 CPUs and 32 GB of RAM per genome and the --skip_tiger flag, skipping integrative conjugative element (ICE) analysis, since we were unable to find the expected reference database or how to generate it. We built a Singularity container that included the databases Beav needs to execute the workflow. Same as with Bakta, 2 genomes (MGYG000298952 and MGYG000298476) were annotated with the --skip-crispr flag enabled due to this portion of the workflow failing.

When performing CPU time calculations, CPU times for Beav, Bakta and PGAP were obtained from the job scheduler (Slurm) reports. Each tool was run on all genomes that it was able to process: a full set of 200 genomes for *mettannotator*, bacterial genomes for Beav and Bakta (194 genomes), and 51 genomes with confirmed species-level taxonomy for PGAP.

**Comparing additional information available for hypothetical proteins**

In the Beav annotation format, additional functional information is included in the “Note” field rather than overriding the “product” field, which results in the same number of hypothetical proteins as in Bakta’s output. In the *mettannotator* GFF file there can also be cases where the product field states that a protein is hypothetical, however, additional tools provide an annotation. To account for this, we looked at the fraction of hypothetical proteins that have additional information from other tools available (Figure 1E in the main text).

We ran both tools on all bacterial genomes in the test dataset (194 genomes). *Mettannotator* was executed in normal mode with Bakta as the gene caller. We considered proteins that have “hypothetical protein” or “uncharacterized protein” as products to be hypothetical. To identify the number of hypothetical proteins that have additional information in the output of Beav, we counted hypothetical proteins that have a “Note” field in the ninth column of the GFF file. To identify the number of hypothetical proteins with additional information in the output of *mettannotator*, we counted the number of hypothetical proteins that have at least one of the following fields in the ninth column of the GFF file: “Ontology_term”, “amrfinderplus_gene_symbol”, “amrfinderplus_scope”, “amrfinderplus_sequence_name”, “cog”, “dbcan_prot_family”, “dbcan_prot_type”, “drug_class”, “drug_subclass”, “eggNOG”, “interpro”, “kegg”, “pfam”, “substrate_dbcan-pul”, “substrate_dbcan-sub”, “uf_chebi”, “uf_gene_name”, “uf_gene_name_synonym”, “uf_keyword”, “uf_ontology_term”, “uf_pirsr_cofactor”, “uf_prot_alt_ecnumber”, “uf_prot_alt_fullname”, “uf_prot_alt_shortname”, “uf_prot_rec_ecnumber”, “uf_prot_rec_fullname”, “uf_prot_rec_shortname”, “note”.

**Assessment of annotation quality**

To assess the quality of annotations produced by *mettannotator*, we chose a well-described capsular biosynthetic locus CR931632 from *Streptococcus pneumoniae* strain 519/43 (serotype 1), which spans a 22 Kb genomic region and consists of 22 genes, including 6 pseudogenes (Bentley et al. 2006).

We identified the location of each of the 22 genes in the genome of *Streptococcus pneumoniae* NCTC7465 strain (RefSeq accession GCF_001457635.1) (O'Leary et al. 2016) by using BLASTN v. 2.12.0 (Camacho et al. 2009). If the hit was truncated or split, we adjusted the start and end positions where possible using multiple-sequence alignment. The positions of all genes in the assembly are shown in Supplementary Table 3.

To compare the quality of annotation produced by *mettannotator* with other tools, we performed annotation of the *Streptococcus pneumoniae* NCTC7465 strain genome using *mettannotator* v1.4.0 in four modes (with Prokka as the gene caller, with Prokka in fast mode, with Bakta as the gene caller, and with Bakta in fast mode) as well as using PGAP v.2024-04-27.build7426, Bakta v1.9.3, Prokka v.1.14.6, and Beav v1.3.0.

To evaluate annotation quality, we compared the performance of each tool in identifying gene boundaries, labelling pseudogenes and identifying the gene name and the product. If a tool called several genes within the boundaries of a single “ground truth” gene, we combined these calls into a single fragmented gene. Fragmented hits only occurred in pseudogenes.

**Supplementary results**

**Annotation quality**

*Gene boundaries*

Gene boundaries were identical in the results of *mettannotator* across all four modes, as well as in the results of Bakta, Prokka, and Beav (Supplementary Figure 5A). This consistency is expected, as these tools use Prodigal as the gene calling algorithm (Hyatt et al. 2010). Differences between the Prodigal-based tools and PGAP only affected pseudogenes, while all remaining genes had identical boundaries across the tools, including the number of bases by which each tool deviated from the ground truth (Supplementary Figure 5B, Supplementary Table 3). When pseudogenes were excluded, all tools identified exact boundaries for 13 out of 16 genes.

*Pseudogenes*

The four *mettannotator* modes produced identical pseudogene results, as expected. *Mettannotator* performed better than Bakta and Beav, which did not detect any pseudogenes, but less well compared to PGAP (Supplementary Figure 5C, Supplementary Table 3). Out of the six pseudogenes, *mettannotator* identified three. *Mettannotator* also erroneously labelled one additional gene as a pseudogene based on the length of the ORF which was shorter than expected. Prokka was excluded from the pseudogene analysis as pseudogene detection is not a part of Prokka’s workflow.

*Functional annotation*

We compared functional annotations of the 16 genes that are not labelled as pseudogenes in the reference (Supplementary Table 3). We first compared the gene names assigned by different tools. Since *mettannotator* inherits gene names from the initial annotation tool (Bakta or Prokka), individual results of these tools do not differ from those of *mettannotator* in normal or fast mode. Therefore, for the purpose of this comparison, we excluded standalone Prokka and Bakta results as well as *mettannotator* in fast mode.

We performed manual curation on gene names which differed from the reference and checked for equivalency or an indication that both genes encode the same or similar product, using UniProtKB (The UniProt Consortium et al. 2023) as reference. We did not identify any cases of a clearly erroneous gene name assignment. PGAP had the fewest gene names assigned (6 out of 16) while *mettannotator* with Prokka as the initial annotator had the most (13 out of 16).

We then compared the assigned products across all tools using the same curation approach described above (Supplementary Table 3). Pseudogenes were excluded as were two genes that did not have product descriptions in the reference.

We did not identify any obviously erroneous product assignments in the results of any of the tools. Prokka was the only tool that did not assign a product to some of the genes, labelling 3 out of 14 genes in our analysis as hypothetical proteins. However, this highlighted *mettannotator*’s performance when transferring product descriptions from databases both in fast mode (without using InterProScan (Jones et al. 2014) and UniFIRE (‘UniFIRE’ 2024)) and in regular mode. In both modes, *mettannotator* correctly assigned product descriptions to the three genes that were labelled as hypothetical proteins by Prokka.

In addition to assigning per-gene annotations described above, *mettannotator* also correctly identified the locus as a biosynthetic gene cluster belonging to the saccharide biosynthetic class.

**Supplementary Figure 1:**

Algorithm to assign functions to proteins labelled as hypothetical by the gene caller. UniFIRE, InterProScan and eggNOG-mapper results are used as sources of functional information.


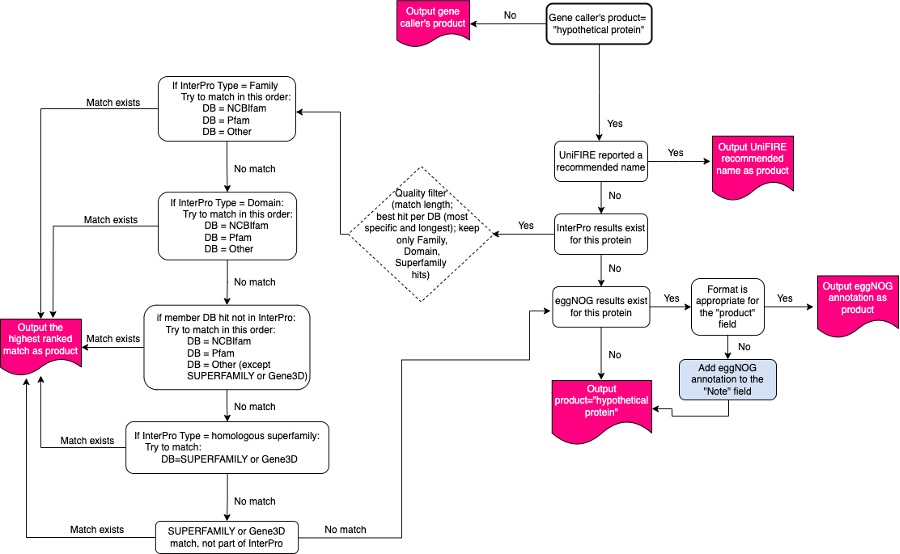


**Supplementary Figure 2:**

Amount of compute time per process when annotating 194 bacterial genomes using *mettannotator* with the --bakta flag enabled.


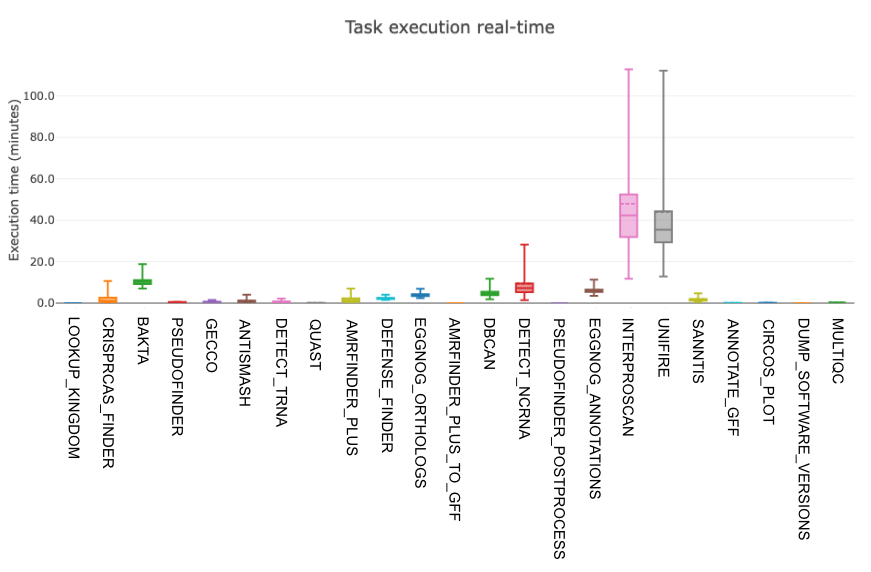


**Supplementary Figure 3:**

Fraction of hypothetical proteins in genomes with poorly known taxonomy. Tools were run on 61 genomes (a subset of the 200 genomes used for benchmarking) that either did not have a species name according to GTDB taxonomy (Supplementary Table 1) or did not use a typical binomial nomenclature indicating the genome likely has not been isolated (for example, “*CAAFZY01 sp900767645*” or “*Phil1 sp004558525*”). *Mettannotator* with Bakta as the gene caller run in normal mode had the least median fraction of hypothetical proteins per genome.


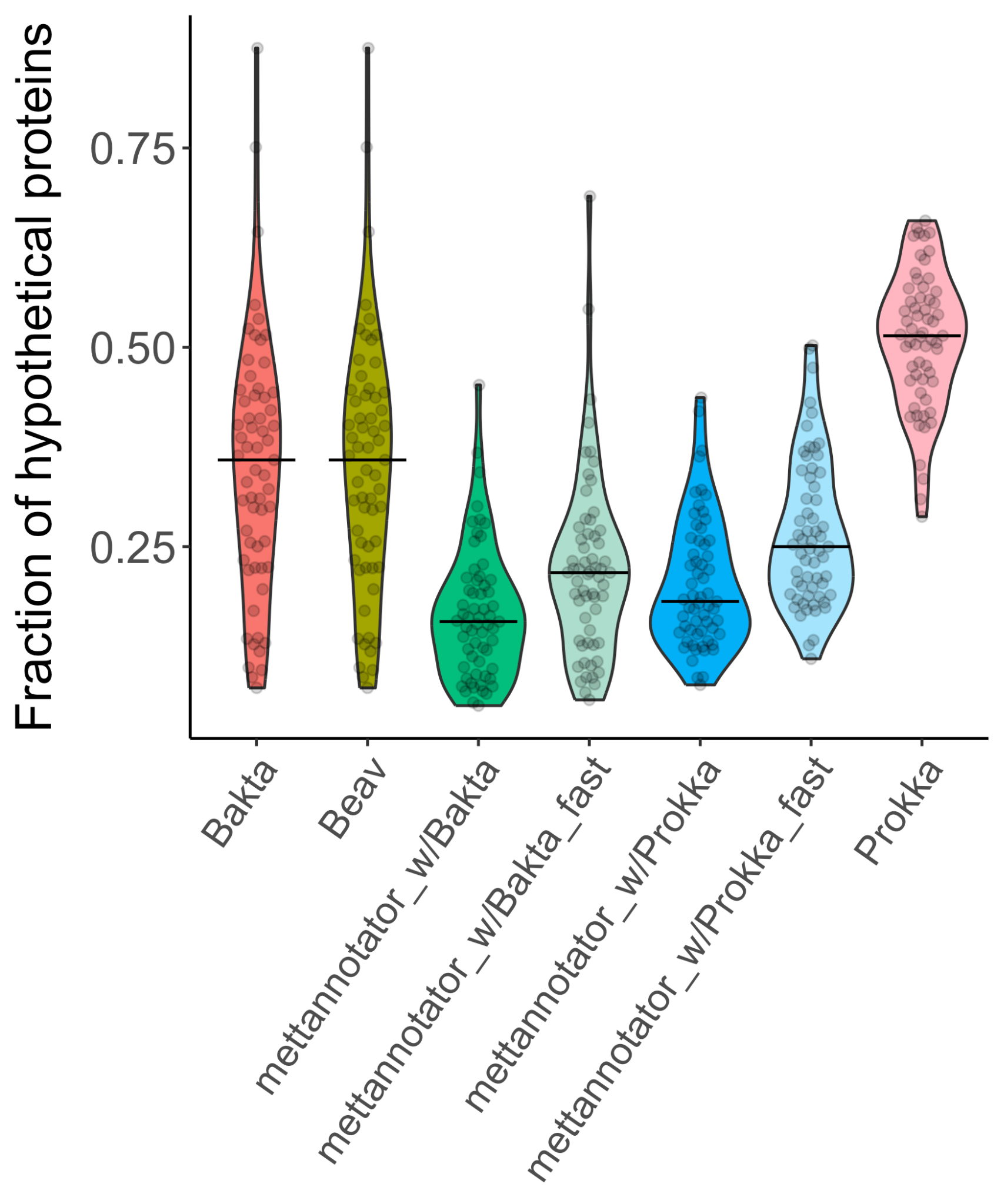


**Supplementary Figure 4:**

The source of product description for CDS in each genome when replacing a protein labelled as “hypothetical” by the gene caller (here, Bakta was used as the gene caller for bacterial genomes). InterPro is used more often to relabel the product than UniFIRE or eggNOG.


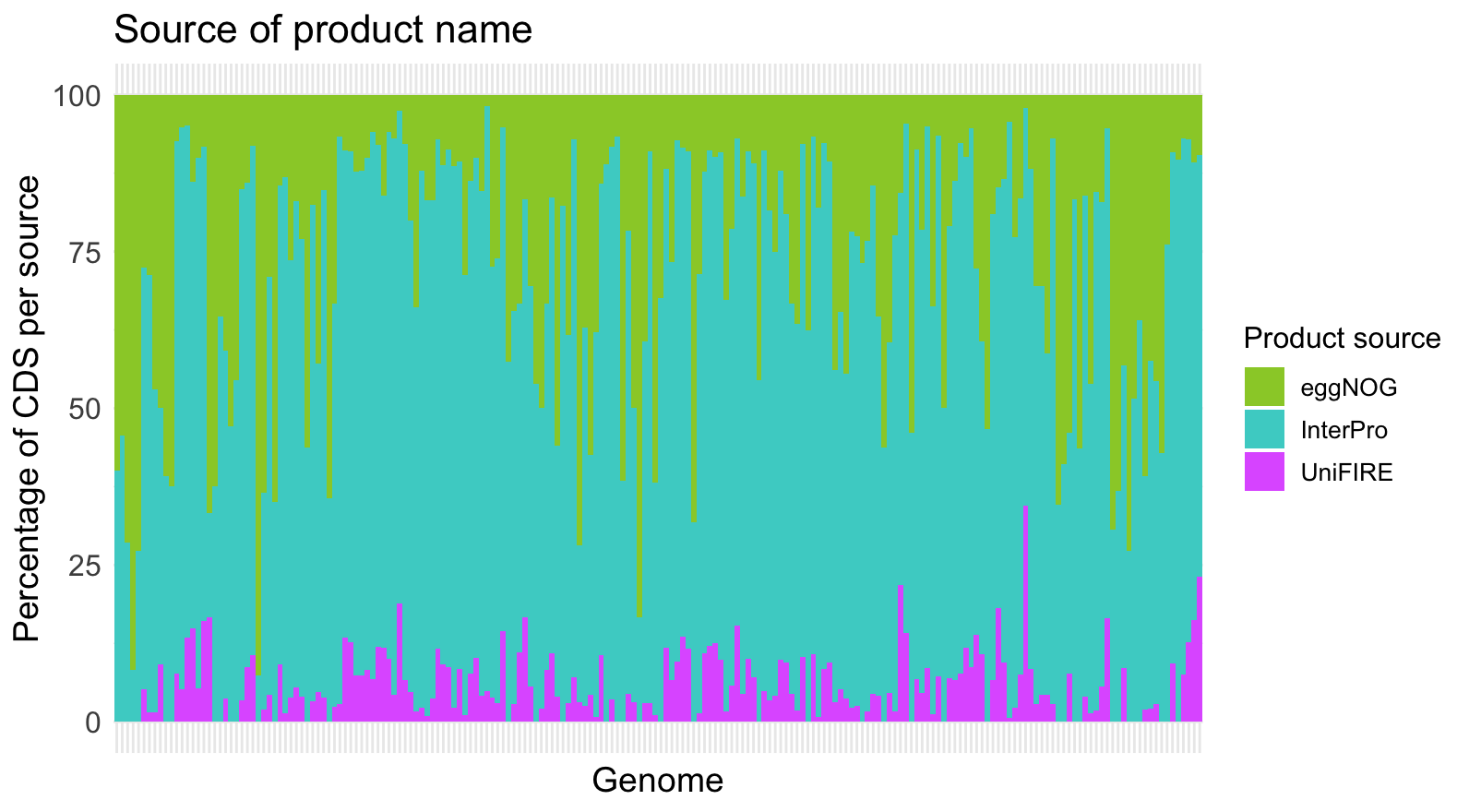


**Supplementary Figure 5:**

Assessment of annotation quality. (**A**) Accuracy of gene boundary prediction. PGAP performed slightly better compared to Prodigal-based tools (*mettannotator*, Bakta, Prokka, and Beav). (**B**) When pseudogenes were removed, the performance of Prodigal-based tools and PGAP was identical. (**C**) Accuracy of pseudogene detection. Beav and Bakta did not detect any pseudogenes while PGAP had 100% specificity and high sensitivity. *Mettannotator* predicted 50% of the expected pseudogenes with 1 false positive.


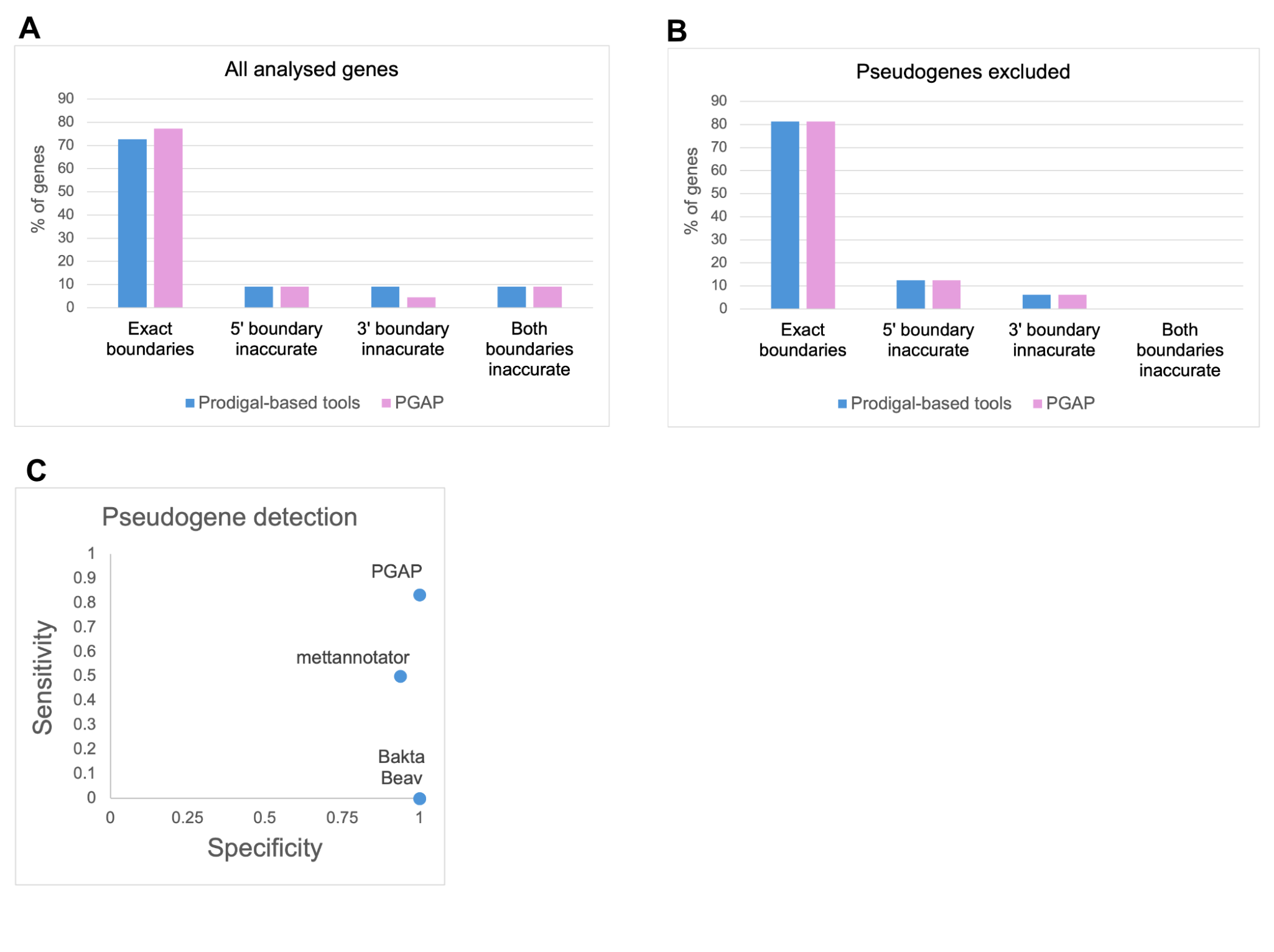


**References**

Bentley, Stephen D., David M. Aanensen, Angeliki Mavroidi, David Saunders, Ester Rabbinowitsch, Matthew Collins, Kathy Donohoe, et al. 2006. ‘Genetic analysis of the capsular biosynthetic locus from all 90 pneumococcal serotypes’. *PLoS Genetics* 2: 0262–0269. https://doi.org/10.1371/journal.pgen.0020031.

Camacho, Christiam, George Coulouris, Vahram Avagyan, Ning Ma, Jason Papadopoulos, Kevin Bealer, and Thomas L. Madden. 2009. ‘BLAST+: Architecture and Applications’. *BMC Bioinformatics* 10: 421. https://doi.org/10.1186/1471-2105-10-421.

Chaumeil, Pierre-Alain, Aaron J. Mussig, Philip Hugenholtz, and Donovan H. Parks. 2020. ‘GTDB-Tk: a toolkit to classify genomes with the Genome Taxonomy Database’. *Bioinformatics* 36 (6): 1925–1927. https://doi.org/10.1093/bioinformatics/btz848.

Haft, Daniel H, Azat Badretdin, George Coulouris, Michael DiCuccio, A Scott Durkin, Eric Jovenitti, Wenjun Li, et al. 2024. ‘RefSeq and the Prokaryotic Genome Annotation Pipeline in the Age of Metagenomes’. *Nucleic Acids Research* 52 (D1): D762–69. https://doi.org/10.1093/nar/gkad988.

Hyatt, Doug, Gwo-Liang Chen, Philip F. Locascio, Miriam L. Land, Frank W. Larimer, and Loren J. Hauser. 2010. ‘Prodigal: Prokaryotic Gene Recognition and Translation Initiation Site Identification’. *BMC Bioinformatics* 11: 119. https://doi.org/10.1186/1471-2105-11-119.

Jones, Philip, David Binns, Hsin-Yu Chang, Matthew Fraser, Weizhong Li, Craig McAnulla, Hamish McWilliam, et al. 2014. ‘InterProScan 5: Genome-Scale Protein Function Classification’. *Bioinformatics* 30 (9): 1236–40. https://doi.org/10.1093/bioinformatics/btu031.

Jung, Jewell M., Arafat Rahman, Andrea M. Schiffer, and Alexandra J. Weisberg. 2024. ‘Beav: A Bacterial Genome and Mobile Element Annotation Pipeline’. https://doi.org/10.1101/2024.01.25.577299.

Li, Wenjun, Kathleen R O’Neill, Daniel H Haft, Michael DiCuccio, Vyacheslav Chetvernin, Azat Badretdin, George Coulouris, et al. 2021. ‘RefSeq: Expanding the Prokaryotic Genome Annotation Pipeline Reach with Protein Family Model Curation’. *Nucleic Acids Research* 49 (D1): D1020–28. https://doi.org/10.1093/nar/gkaa1105.

O'Leary, Nuala A., Mathew W. Wright, J. Rodney Brister, Stacy Ciufo, Diana Haddad, Rich McVeigh, Bhanu Rajput, et al. 2016. ‘Reference sequence (RefSeq) database at NCBI: current status, taxonomic expansion, and functional annotation’. *Nucleic Acids Research* 44 (D1): D733–D745. https://doi.org/10.1093/nar/gkv1189.

Parks, Donovan H., Michael Imelfort, Connor T. Skennerton, Philip Hugenholtz, and Gene W. Tyson. 2015. ‘CheckM: Assessing the Quality of Microbial Genomes Recovered from Isolates, Single Cells, and Metagenomes’. *Genome Research* 25 (7): 1043–55. https://doi.org/10.1101/gr.186072.114.

Schwengers, Oliver, Lukas Jelonek, Marius Alfred Dieckmann, Sebastian Beyvers, Jochen Blom, and Alexander Goesmann. 2021. ‘Bakta: Rapid and Standardized Annotation of Bacterial Genomes via Alignment-Free Sequence Identification: Find out More about Bakta, the Motivation, Challenges and Applications, Here.’ *Microbial Genomics* 7 (11). https://doi.org/10.1099/mgen.0.000685.

Seemann, Torsten. 2014. ‘Prokka: Rapid Prokaryotic Genome Annotation’. *Bioinformatics* 30 (14): 2068–69. https://doi.org/10.1093/bioinformatics/btu153.

The UniProt Consortium, Alex Bateman, Maria-Jesus Martin, Sandra Orchard, Michele Magrane, Shadab Ahmad, Emanuele Alpi, et al. 2023. ‘UniProt: The Universal Protein Knowledgebase in 2023’. *Nucleic Acids Research* 51 (D1): D523–31. https://doi.org/10.1093/nar/gkac1052.

‘UniFIRE’. 2024. https://gitlab.ebi.ac.uk/uniprot-public/unifire.

von Meijenfeldt F.A.B., Ksenia Arkhipova, Diego D. Cambuy, Felipe H. Coutinho, and Bas E. Dutilh. 2019. ‘Robust taxonomic classification of uncharted microbial sequences and bins with CAT and BAT’. *Genome Biology* 20:217. https://doi.org/10.1186/s13059-019-1817-x.
